# Age tourism: going beyond health and “triple S” tourism toward a new request of journey

**DOI:** 10.3389/fsoc.2024.1395405

**Published:** 2025-02-25

**Authors:** Letizia Carrera

**Affiliations:** Department DIRIUM, University of Bari Aldo Moro, Bari, Italy

**Keywords:** ageing, age tourism, tourism market, experience, local heritage

## Abstract

For many decades, the large part of developed countries has been experiencing the progressive ageing of their populations. This quantitative change is also accompanied by a qualitative shift in social representations of the *third age*. Within these changes, a fundamental role is played by the desire to experience opportunities for socializing, leisure, and culture that can shape a new and more complex concept of well-being. Within this context, tourism experience plays a crucial role. The focus is therefore on the new characteristics of senior tourism and the conditions under which it can represent a full and satisfying experience, going beyond the classic offerings of “sun, sand, and sea” or medical tourism. To this end, a qualitative study was conducted, revealing a typology of individuals that can serve as a useful reference for the tourism market to diversifying its offerings.

## 1 Introduction: the *new* older

From many decades, the large part of developed countries is facing the progressive ageing of populations. Today, more than 8% of the world’s population is over 65 years old (Ritchie and Roser, 2019), and the number of people reaching the age of 100 is at the highest level in history (Robine, 2021). The Italian population, like those in other Western nations, is becoming increasingly older as a result of both the decrease in the number of new births and the lengthening of life expectancy. This quantitative change is also aligned with a qualitative one. At least some of them are moving away from traditional representations of old age toward a new idea of the ageing process, starting to reclaim full recognition of their needs and desires for culture, leisure, and opportunities to socialize. The demographic trend in ageing is a widespread phenomenon which implies wider changes, and it has been argued that these older segments of the population will not only live longer, but also be healthier and more active, have more income and time, and moreover expect a higher quality of life than similar age groups in previous decades. This substantial socio-demographic change is producing and will produce in the close future important consequences and represents a fundamental task for national and territorial welfare public policies.

These changes reinforce wider opportunities to adopt a greater awareness of the extreme diversity of experience within the classification “older people” and a more sophisticated concept of well-being as the synthesis of physical and mental health. According to Rowe and Kahn’s successful ageing model (1997), going beyond the link with diseases and disabilities, increasing mental functioning and general wellness have been recognized as factors that delay ageing and lower the age one feels (WHO, 1999; WHO, 2015a; Stowe and Cooney, 2015). And for this reason, in accordance with the World Health Organization’s global report on ageing and health (WHO, 2015b), it is necessary to ensure the conditions for an healthy life. So, it is necessary to adopt guidelines for targeted policies that national governments and territorial institutions should develop to increase the quality of life for older individuals and improve their overall well-being because the increasing life expectancy does not automatically lead to an active ageing process (Caradec, 2001a, 2001b; Moulaert and Paris, 2013; Bai, 2014; Buffel and Phillipson, 2018; Cotterel and Buffel, 2018; Carrera, 2020; Wang et al., 2024). In this perspective, the ageing of the population presents a fundamental challenge for public institutions. Starting from the complexity of ageing issues and the plurality of rights that should be granted to older people: to mobility, to care, to safety, to proximity, to a habitat adapted to their changing needs, to well- equipped public spaces, to urban greenery, to cultural occasions accessible and widespread throughout cities, to leisure and opportunities to socialize (Carrera, 2020).^1^ These rights can be synthesized under the “right to the city” which Henri Lefebvre (1967) wrote about more than 50 years ago and which, recently, Harvey (2016) rethematized. This concept can be translated into the right to live in an inclusive and accessible urban habitat (WHO, 2007), rich in opportunities for social interaction and high levels of well-being. The new and varied forms of the elderly condition are influenced not only by the material dimension of space and its infrastructure but also by the immaterial dimension of the conditions that ensure opportunities for an active life.

In this perspective, a central role is played by tourism capable of synthesizing cultural needs, leisure, and also socialization. However, it cannot be forgotten that the new elderly (Carrera, 2020) require a rethinking of the tourism offer itself, which must be able to go beyond health tourism (Letunovska et al., 2020; Yorulmaz, 2019), and/or the traditionally targeted tourism aimed solely at the elderly with sun and sea (the triple S) to respond to more complex demands for new forms of travel and its potential for experience. The profound change in the condition of elderly individuals also requires, with reference to this specific area, recognizing the complexity and differentiation experienced by these individuals. Specifically, this is the subject that has been intended to investigate through a qualitative study aimed at exploring the individual contextual dimensions influencing the tourism experience of elderly individuals.

The ageing of populations in developed countries poses new challenges not only for healthcare systems, but also for tourism and recreation. This new and more complex attention to the ageing process has increased the number of studies on old age and, within them, those on health tourism (Letunovska et al., 2020), geriatric tourism (Büyük and Akkuş, 2022), and, more generally, third age tourism. In this changed scenario, the tourist experience, urban and extra-urban, can play an important role by combining the needs of culture, leisure, and opportunities to socialize, as well as that core desire for experiences increasingly important to many older people.

## 2 The ageing process and new representations of the third age

Italy, in line with a European and global trend, is an increasingly old country. Older people over 65 years number more than 14 million at the beginning of 2022, 3 million more than twenty years ago. In a few decades this number will grow to 19 million, confirming this increasing trend. The direction of the progressive ageing of the population does not differ significantly from the European averages reported by Eurostat, which confirms the evidence of a widespread and structural process that needs to be confronted and that has been called one of the most important challenges of our times (Moulaert and Paris, 2013; Attias-Donfut et al., 2005). “Policies to meet the challenge of a growing, healthier and more active elderly population—based on the view of the ageing of society as an opportunity to be utilized—automatically benefit the individual ageing person, materially and otherwise. Similarly, any effort to ameliorate the quality of life for the elderly, and to meet their diverse social and cultural needs, enhances their capacity to continue interacting with society” (United Nations, 1982, p. 16). This is explained by the connection between the quantitative changes and the qualitative ones related to new different social representations of old age and in the meaning attributed to being and becoming older (Laslett, 1991; Eurisko, 1999; Phillipson, 2020; Facchini and Rampazi, 2006). Indeed, in recent decades being older has begun to be considered no longer a mere residual condition compared to the active condition of adult workers, but rather a real new phase full of opportunities and possibilities, even if with limits and difficulties (Carrera, 2021). It is essentially a new age of life for humankind (Apriceno and Levy, 2023; Scortegagna, 1998). The third age is the “third time” discussed by the anthropologist Ravera (2023), who considers old age as the time for learning *how to change* by adopting new logics, going beyond those of efficiency, competition, and speed, too often taken for granted. It is, or rather may be, the time for the invention of new models, new values, and new ways of living. Society is facing a new generation of older people outside the bounds of every cultural paradigm known until now, a sort of new species of subjects. To prevent the quality of life of the elderly from depending exclusively on their economic, social, and cultural resources, social and regional welfare as the community welfare too can play a key role in responding adequately to these new and different needs by planning structured interventions guided by objectives that ensure the conditions for healthy and active ageing and a meaningful life (WHO, 2017; Crocker et al., 2024; Wang et al., 2024; Portegijs et al., 2023; Kim et al., 2023). If welfare is absent or even just inadequate, the consequence would otherwise be to deny to a large segment of the older population the right to a substantial equality of opportunities (Nussbaum, 2002; Sen, 1986, 1992).

It is equally important to remember that dealing with ageing implies an awareness of the extreme diversity within the “old age” classification (Gilleard and Higgs, 2002). This will all differ widely in terms of lifestyles, experiences, needs, desires, and projects (Alwin and Hofer, 2011). Old age can be a phase characterized by fullness and self-realization, by new plans and a renewed search for meaning, or instead, by fragmentation, loss of self, loneliness, and isolation, even marginalization and social exclusion depending on personal and social factors and resources (Carrera, 2020). These individual factors intersect with context-specific ones and vice versa and together can heavily influence the differentiated levels of quality of life among older people. Among the factors attributable to the individual subject are the state of health, the level of autonomy, the endowment of economic and cultural capital, and the quality of family and friend networks. Some of the context-specific factors include the quality and accessibility of public spaces, the state of public transportation, the provision of green spaces and well-equipped parks, the perceived level of safety in the city, and opportunities for culture, leisure, and socialization. Within this framework, tourist proposals—broadly speaking—can play a fundamental role.

This complex combined process becomes even more evident when looking at the *new older* (Carrera, 2020), those who perceive themselves in a different and transformed way, increasingly capable of desires and life planning, and searching for experiences and existential fullness. But, also for *other older people*, those who are closer to traditional representations of ageing, the role of social opportunities is fundamental to identifying and to pursuing new paths and new possibilities for active ageing (Wang et al., 2024; Portegijs et al., 2023; Bledowski et al., 2011; Cotterel and Buffel, 2018). However, despite the persistence of the traditional pattern of representations of old age, the new ones focused on a different social and cultural vitality are becoming more and more consolidated. This has generated and will continue to do so in the near future, the need for integrated public and private welfare services in order to create the conditions for an active life, not only free of disease but also full of well-being. If public welfare often seems to still have difficulties interacting with older citizens (Poli, 2012; Carrera, 2020), the market has already recognized them as carriers of a purchasing power and of a new and strong desire for leisure and socialization (Cottardo, 1996; Long, 1998). Consequently, it has started to offer them specific products like cruises and trips, home renovation ideas, and other opportunities designed for this specific target group of consumers aligned with the desire of older people to live their lives to the fullest. Within the market offer, population ageing is a new demographic challenge also for tourism proposals, which can respond with new types and forms of tourism, as well as new technologies of service.^2^ Within this larger process of a growing number of requests for experiences and emotions, the tourist offer represents a synthesis of these complex desires and a tool to satisfy the *new* needs of the *new* older.

## 3 Age tourism and the desire for experiences

The desire for travel and tourism is increasingly a quest for experiences, emotions, intangible, authenticity (Amendola, 2013). The usual destinations founded on the “triple S”—sun, sea, sand—have needed to be profoundly rethought and redesigned on their differentiated targets and especially to improve the attractiveness of their traditional offers. The tourism sector has registered a steady development of new products based on natural, business, leisure, anthropological, gastronomic, and spiritual offers. More and more, tourist travel is becoming a sort of *intellectual project,* not only an escape from everyday spaces, but a unique life experience and emotions that places may guarantee (Cockerell, 1993; Costa, 1993; Savelli, 2004). Within this “postmodern model of tourism” (Sutton and House, 2003) the imaginary has, more than in the past, a fundamental function^3^ and heavily influences the perceived attractiveness of places (Corbisiero, 2021; Colleoni and Guerisoli, 2014; Aime and Papotti, 2012; Bouchard, 2014, 2018; Avallone, 2019; Marra and Ruspini, 2010; Menegaldo and Menegaldo, 2007; Salazar, 2011).

The search for the meaning of places, for the emotions they may arouse, for the experiences they may induce, transforms the journey into an opportunity for intimate and transformative cultural experiences that, increasingly, involve older people, too (Carrera, 2020; Clement, 2019; Vergunst, 2008; Sgroi, 2002; Nicholson, 2001). There is, indeed, evidence of a strong current and quite global increasing demand for senior travel (Fleischer and Pizam, 2002; Echtner and Ritchie, 2003; Handler, 2014; Losada et al., 2016). These senior travel experiences have moved more and more away from the classic needs related to the simple “triple S” experiences or to health tourism, and toward trips able to ensure a state of well-being, synthesis of “body and mind” in a holistic sense. Within this perspective, tourist experiences, indeed, are perceived as gratifying and useful for seeking intellectual stimulation and learning, developing new skills, maintaining physical fitness, relaxing, meeting new people and socializing, and visiting friends and relatives^4^ (Horneman et al., 2002; Kim et al., 2003; Bartoli, 1994).

Obviously, geriatric tourism and older care tourism continue to represent an important part of senior tourism offers. Geriatric tourism brings together the health and tourism sectors by combining alternative and complementary medical procedures and tourism activities, which are considered beneficial for ensuring and maintaining health and well-being among older people (Sert, 2019; and, specifically about the term “geriatric tourism,” Tsartsara, 2018; Yildiz et al., 2013; Yorulmaz, 2019). But, even if the ageing process implies a fast or slow constant deterioration in health and physical abilities (Hsu et al., 2007; Dilekçi et al., 2020), studies show that older people feel happier and more active when they participate in cultural and social activities like tourism (Hsu et al., 2007). These activities have a positive impact on their mental and physical wellness, gaining support from alternative and complementary treatments and professional medical interventions, and can have a deep impact on the maintenance of physical as well as mental health (Dilekçi et al., 2020). Bartoli (1995) wrote that the trip helps to alleviate psychological problems such as the loss of professional and social roles, loneliness or apathy and, from this point of view, the journey can be called the *real medicine* for older people. These kinds of tourism fall into two macro groups: (a) medical services for diagnosis and treatment and (b) auxiliary services for health promotion, including beneficial thermal facilities. In this perspective, this includes some activities related to traditional and complementary medicine, thermal tourism, and others related to complementary and alternative medicine (İçöz, 2009; Şengül and Bulut, 2019).

However, tourism for elderly individuals is increasingly moving away from health-related issues and toward an innovative and more complex concept of well-being. These new types of tourism proposals aimed at elderly individuals present both tourist packages dedicated to meet the specific needs of travelers and older tourists, and others which are instead generic and undifferentiated but, for their characteristics, also usable by these specific groups. Both of these types are referred to a large part of older subjects, who live old age as a new and different experience, “reducing and a little also accepting its disadvantages, and learning to exploit its advantages with philosophy and a spirit of curiosity” (Levi, 1998), and who view holidays as an important opportunity to realize rich and rewarding experiences. The second kind of tourist offer shows how at least some older tourists’ requests are even more distinct from the past because elderly not only claim their *right to tourist experiences* but also ask to be able to take advantage of the “normal” ones that they consider “not ghettoizing,” even if they require specific preparations. So, we are facing a new phase, *age tourism 2.0* (Carrera, 2022).^5^ This means that the requests tend in the direction of trips accessible also for elderly subjects but not exclusively geared for them so as to be able to make richer experiences at the level of social and cultural encounters.

In terms of supply, however, there are not many tour operators who have already equipped themselves for non-specific offer, so there are still few inclusive packages proposed *also* to older people. Instead, there are still many sites that use the label “holidays for older” even if they go beyond the classic destinations and offer relaxing and intense atmospheres and activities, but without requiring a high level of physical exertion. In the case of both dedicated packages and generic ones, age tourism cannot avoid having some specific features. Despite the extreme variety of tourist offers addressed to the elderly as a specific segment of travelers, there are some common features such as short trips, transfers and animation activities alternating with periods of rest, choice of climatically suitable periods, provision of a constant presence of reference staff for the various needs or problems that may arise, adequate modes of transportation, and suitable and comfortable accommodations. One of the most interesting services offered to these tourists is the telemedicine provided by the accommodation facilities, not reserved for elderly tourists, but that pay particular attention to them (Stončikaitė, 2022, p. 336).^6^

Thus, population ageing is a new demographic challenge in society to which the tourism industry will have to respond with new types and forms of tourism, new technologies of service. E.g. the increase in the segment known as “third age” tourists, the formation of a new segment of “senior tourism,” the creation of high-tech hotel and resort complexes with a wide range of geriatric care options to promoting active ageing, and, of course, the widespread adoption of the technology, telehealth and telecare, that will support the aspirations of older people to travel and continue their active lifestyle (Nikitina and Vorontsova, 2015; Klimova, 2017). These proposals are connected with the general characteristics of third age tourists are listed as follows (Huang and Tsai, 2003; Möller et al., 2007; Zsarnoczky, 2016): (i) having sufficient disposable income; (ii) being mostly women; (iii) prioritizing safety; (iv) tending to prolong their stay in destinations; (v) being able to travel all seasons because of free time; (vi) being considered “curious” tourists; (vii) caring about communication; (viii) being influenced by each other’s advice; and (ix) caring more about access than transportation. Specific and different perspective is the offer specifically aimed at elderly people who are not self-sufficient that moves in the direction of social tourism—for disabled older people with temporary or permanent impediments—toward the objective of guaranteeing for all subjects the right to travel, in line with the requirements of the 1996 Montreal Treaty.

This extreme diversification in terms of tourist supply and demand and its potential developments clearly show how the reflections on the theme of senior tourism linked with the specific features of the ageing process and its new, complex, and differentiated specificities, require targeted analysis and research (Kim et al., 2015; Horneman et al., 2002). Until now, the analysis conducted have highlighted the importance of applied tourism research, providing insights into and evidence about the changing nature of senior tourism within the tourism sector as a whole (Moscardo, 2006, p. 30).^7^ According to Pearce “it is a part of the spirit and mission of tourism research to use academic and analytical observation and skills to assist emerging business developments while at the same time enhancing our understanding of tourism phenomena” (1999, p. 36).

## 4 Older people tourism desires between healthcare travels and experiences journey: a qualitative study

In order to investigate the characteristics of choices and tourist experiences among older subjects, a focused research project was conducted using a qualitative methodology. The sample of 102 subjects, aged between 65 and 85 years old, of both genders, resident in Puglia, was identified. These subjects were randomly selected, but with attention to ensuring that the various socio-demographic variables considered, -such as age, gender, income level, and educational background -, were adequately represented. To these subjects were addressed semi-structured interviews centered on two thematic topics: their travel desires and their underlying motivations; their behaviors and concrete experiences on actual trips. The semi-structured interview method^8^ was chosen because the aim of investigating not only behaviors but also attitudes and orientations would not have been adequately captured through the use of a questionnaire.

Based on the grid thus obtained, the interviewed subjects have been identified as carriers of a direct experience about the observed phenomenon, so the unit of analysis in the research coincides with the unit of detection (Acocella and Cataldi, 2020, pp. 113–115). The sample is thus composed according to the main socio-demographic variables taken into consideration:

- gender: 59% women – 41% men;
- degree: 42% bachelor’s degree and over – 31% upper secondary – 20% lower secondary – 7% primary school (elementary);
- residence: 35% Bari – 21% Taranto – 17% Foggia – 12% Lecce – 8% Brindisi – 7% BAT;^9^
- declared income level: 40% high – 36% average – 24% low.

The interviews, translated into protocols, were treated to a thematic analysis that, crossing the two thematic axes taken into account^10^—(1) high/low desire for travel and (2) high/low concrete experience of travel—has brought out four different types of representations and behaviors. From the intersection of the two dimensions investigated and based on the results of the interviews, it was possible to obtain a grid that allowed the identification of four main types of elderly tourists: (a) *travelers*; (b) *tag alongs*; (c) *stand by*; (d) *motionless*.

(a) *Travelers* are people with a high level of desire to travel and a high frequency in terms of concrete behaviors. The subjects of this type have some common characteristics: they are both women and men with a medium and high degree of study, mostly residents in the cities, with a good economic income, membership in associations, and included in a medium-large friend network. Regarding the motivations underlying their wishes and experiences of travel, however, there are some marked differences that suggest the need to differentiate three subtypes: *pure travelers*, *tourists*, and *health conscious*.

The *pure travelers* are those who affirm their love for the cultural and emotional experience that journey guarantees them. They have a long history of trips that continues, within the limits of their physical autonomy. They look for less well-known destinations, acquire information for themselves online, and travel preferably in small “already run-in” groups. They are endowed with a high cultural capital and a discreet or high economic capital and choose “comfortable” but interstitial solutions compared to the more classic destinations, and each trip lasts about a week. They choose to know the cities by walking through them, albeit with the eventual limitations related to their physical condition. They strongly underscore how the journey represents an opportunity to experience places and a cultural enrichment, that the destinations sought are those less well-known because that makes them more “authentic.” They do not mix their journeys with health-related ones, which they also seek but do not consider a “real journey.” They often travel independently, building their own itineraries because they consider the offers specifically aimed at the elderly “banal and poor.” They are moreover self-directed tourists able to organize their own travels—*Fit (Free Independent Travel)*—without the mediation of travel agencies, even if some companies, however, are beginning to offer services to assist independent travelers. Very often older tourists even tell travel agencies that offers that are specifically proposed to the elderly are limited and even boring and ghettoizing.

“I travel whenever I can. I wished to do it even when I was younger, but with the family, I have three children, work, and not much money… But now, finally, I travel a lot. Of course, small trips of maximum 3–4 days because I get tired easily, and then my shoulder hurts, but I travel. When I was a teacher, I always taught my children the importance of travel. I do not like elderly trips; it’s nice when they are mixed, talking about everything, meeting interesting people, and seeing more interesting places. (…) The only problem is that I am alone, and if I do not find someone to travel with me, I give up. It’s bad to remain alone. It’s like a snake biting its tail, the more you are alone, the more you remain alone (…) If there were better offers, I would travel more. It’s not a matter of money but of loneliness” (woman, 84 years old).

The second subtype, the *tourists* presents a medium number of travel experience (no less than two a year), traveling preferably in organized groups, and relying on travel packages and agencies specializing in *silver tourism*. This step is fundamental to be able to enjoy specific offers suitable for their health, and they look for constant support during the trip to enjoy places without risks. They have an average level of cultural capital and a medium-high economic availability. They look for attractive destinations by accepting proposals from agencies or friends who have already made a similar journey. They choose transportation solutions such as busses or airplanes for the most distant destinations and use organized tours to cross the city, “so you can see as much of a city as possible.” For them the trip is an opportunity for leisure and to visit new places, especially with friends. These subjects look for opportunities for new experiences along the way, but the dimension of socialization and fun seems paramount. They clearly state that they will continue to travel as long as they can and complain about the limited variety of offers dedicated to their age.

“I have worked all my life, now I want to enjoy it with my wife. We also travel three times a year; sometimes we take a grandchild with us. We like to do organized trips so we do not get tired because they know how to organize the trip and the days, and the tours on organized busses are ideal; you see everything and do not get tired. It’s fun, and at seventy, you need some tranquility and fun. The place is less important than the group we travel with, but now we are a small group of people who travel together” (man, 71 years old).

The *health conscious* are subjects who travel a lot for health issues: spa stays, seas and hills in places equipped for a specific type of stay. They are both men and women with a medium economic level and hold a secondary or high school diploma. They often travel in pairs but sometimes use the welfare offers of their work companies. For almost everyone the dimension of self-care linked to physical well-being is prevalent, but these trips also have the function of making them relax and let them live a few days off their regular routines. For *health conscious* subjects travel is not so much the search for a new place to get to know as the opportunity to escape from everyday life and to have some fun.

“I often travel because as a retiree, my company offers thermal stays, spas, and even sports weekends, certainly suitable for seniors. For me, it’s great, even when my wife does not want to come, I go alone, have fun, and relax; then I come back feeling better, even in terms of health. I have heart problems, and traveling is good for my health and mind” (man, 78 years old).

(b) The *tag alongs c*ombines a low desire to travel with frequent experiences. They let themselves be convinced by their partner and friends, and live the journey as an occasion to socialize rather than to get to know new places. They move exclusively in groups, more or less large, entrusting the organization of the trip “to those who care and know better how to disentangle within this experience.” They are subjects, in the sample considered mainly women, with a medium degree of cultural and economic capital, who do not have a great habit of traveling, which they describe above all as a great discomfort and which only serves to please their partner and their friends. They agree to go only if they know at least some members of the group with whom they will have to travel.

“I live in a village, and I’m really a village woman. I’m not ashamed to say it. At seventy, I do not change anymore; my husband wants to travel, but I’m fine at home. I have my things, my neighbors, I have a nephew. I’m fine here. But sometimes I have to make him happy, so I let him convince me to go. It’s not that I do not enjoy it, but when I’m away, honestly, I cannot wait to be back home. But sometimes we are a small group, and it’s easier to have fun because I know who to talk to” (woman, 68 years old).

(c) The *stand by* is someone who declares a great interest in travel experiences but undertakes it only occasionally, complaining about the scarcity of economic resources or, above all, not knowing with whom to travel. They are subjects, generally women, placed outside of structured networks of friends, such as parish or associative groups, and who experience the lack of travel as a loss of opportunities that “never come back.” Endowed with a medium level of cultural and economic capital, they would be available to deal with travel if it were adapted to their health conditions, not too expensive, and, above all, they could find someone to be with. For these subjects, the importance and the burden of relational poverty appears to be marked and undermines the possibility of realizing experiences that they would like. The perceived feelings of loneliness are underlined by their statement that “knowing even just one person” would be enough to not have to give up traveling. These subjects experience the absence of a travel habit as a “painful lack.”

“I would always travel, to spend some time with my children who live far away, but travel is a beautiful thing. I would like to see many places; there is so much to see in the world. But instead, I hardly ever travel. And how can I… (…) Money is scarce, and every trip costs, but especially with whom do I travel??? Being alone is so awful. Once I took a trip to buy pots that they proposed to me in the supermarket, but I regretted it. They were all in groups, in couples, and even though I am elderly, the wives did not want me and did not include me. One evening I cried. The more you are alone, the more you remain alone! (…) The parish here only thinks about masses and the Rosary. They do not do anything; they do not understand the needs of elderly people like me who are alone. And since my husband died, I’ve been alone” (woman, 73).

(d) Finally, the *motionless* are subjects with a low, in fact almost zero, desire to travel and practically no experience in this regard. They have a low cultural capital and a low or simply average level of economic capital, and consider travel something that is not suitable for their age, regardless of their level of physical autonomy. They are not interested in doing something that they perceive as a completely useless—even dangerous—effort, rather than a rewarding and enriching experience. They declare a low level of relationality, which takes shape especially in the small size of their family networks and in the substantial absence of a friend network, which in some cases reflects a marked condition of relational poverty. Unlike the previous type, they do not seem to suffer from this condition, because they consider important and useful only those trips made for health reasons. They embody the more traditional representation of old age marked by a short and low-quality network of relationships, which especially for women results in a high level of domesticity, involving a higher risk of lineless and social isolation.

For this type, as well as for the previous one, the limits connected with mobility difficulties are particularly significant. Not driving or not driving anymore, their travel possibilities are absolutely reduced. For those who have this low social, cultural, and economic capital and who live in peripheral areas of their cities or in small towns, the lack of organized tourist opportunities plays a decisive role in defining the generally limited options available to them.

“I’m old, where should I go at this age??? My children travel, and then I see the photos, I’m fine like this. Hey, I’ve never traveled even when I was young, and honestly, I do not have the desire to. Then even if I wanted to take a trip, I would not even know how because I do not know anyone who organizes trips, and those from travel agencies I know they do things that are too expensive and tire you out. Maybe once, especially if it’s something for seniors, I could consider it” (woman, 75 years old).

## 5 Conclusion

As historian Andrea Riccardi writes, “the elderly are the continent to be explored” (Riccardi, 2013, p. 8) and, it could be added, to rethink design. Having ensured that life expectancies have been prolonged, we need to think about how to fill those years with meaning starting from reflection on new ways of traveling, of exploring, of using their time, of dwelling. As Lidia Ravera writes, actually referring in a particular way to women, but in a way that absolutely could be extended to older subjects as a whole, “the Third Time of life is a good time to practice changing” (2023, p. 12). The elderly are increasingly ready to prolong their healthy and active lifestyles through the search for cultural, leisure, and social opportunities, and it can find in tourism experiences a synthesis of those opportunities, increasingly felt not only as mere desires but as real needs (Kim et al., 2003).

The desire for travel is an important part of this “new age of desire” (Ravera, 2023) to which, moreover, the market, as noted, looks with increasing interest in the so-called *silver economy*. So, the *new elderly* find in tourism an important opportunity to respond to that change in representations and self-representation discussed at the beginning. Tourism, both with its dedicated proposals and with its more generic ones, represents a field of great interest and a challenge that is still wide open. Under certain conditions, in fact, a positive impact can be generated by the possibility of enriching the experiences of the older people and furthering regional economic development, through seasonally adjusted solutions.

And in this sense, senior tourism, with its characteristics of slow tourism, potentially less seasonal, seeking not only art cities but also more intermediate places capable of offering opportunities for experiences and emotions, can be effectively linked to virtuous strategies for non-myopic local development (Ritchie et al., 2003). This means that if the tourism sector manages to tailor itself to the specific needs of these types of tourists and travelers without falling into the trap of age-related stereotypes, senior tourism can be a virtuous synthesis of quality of life and well-being and economic impact on a sector that has shown to be driving for the local economy toward sustainable development (Sakai et al., 2000; Fleischer and Pizam, 2002; Huang and Tsai, 2003; Kim et al., 2003) The changing age patterns of consumers will require revision of the strategic approaches to the segmentation of consumers in the sphere of tourism and recreation. “Researchers and tourism practitioners need to be cautious about adopting age-based stereotypes, as an increasingly common theme in service quality studies is that of resentment of negative ageist stereotypes. However, practitioners also cannot ignore the real constraints that result from physical ageing, and operators may need to develop medical services as a part of their tourism products. A major challenge for future research in this area is to examine the facilities and services that appeal to seniors but do not create a sense of negative isolation” (Moscardo, 2006, p. 39).

Indeed, despite the limitations of the qualitative research conducted, one of the obstacles to translating the desire to travel into a concrete experience appears to be the absence of friends with whom to undertake it. This factor seems to have a particularly significant impact, especially concerning women (Small, 2003; Carrera, 2020). It has confirmed the profound variety in the desires and behaviors of older subjects in relation to travel choices, but also how much the condition of health and even the level of self-autonomy are less relevant than the cultural capital and the presence of family and friend network and opportunities offered by associations present in the region.

Moreover in women words, the desire for travel is limited, if not denied, both by poverty or the lack of opportunities for travel and, especially in the words of women, by the limited nature of their social networks. Not having someone with whom to realize the travel experience becomes an extremely effective and high-impact hindrance. This condition easily becomes a circular process that excludes, especially women with weak and short social networks, from further opportunities for widespread social interaction.

Recognizing the value of tourism for counteracting cultural and, above all, relational poverty and the corresponding loss of vitality, and for improving the quality of life and the well-being of older people, it seems important to monitor and encourage the provision of affordable cultural and social services, both public and private ones, in every part of the city. This objective can be more effectively pursued by developing the quality of tourist offers not focused on older people and by welcoming older consumers’ expectations, going even beyond those toward a project of widespread growth in living conditions and opportunities for the elderly in different regional habitats.

In this context, public policies can play a key role both in incentivizing and fostering a tourism market that operates under certain conditions and in contributing to the creation of places and opportunities for social interaction across territories to facilitate the formation of groups of individuals who can shape their travel desires together. These reflections, therefore, open up to new forms of research related, among other things, to the role of local administrations and urban policies in promoting a widespread culture of travel and its concrete potential, recognizing them as central to the goal of the well-being of elderly individuals.

## Data Availability

The raw data supporting the conclusions of this article will be made available by the authors, without undue reservation.

## Footnotes

^1^It is necessary to reconceptualize quality of life in terms of an interrelation between urban spaces or, more generally, urban habitat, and the principles of social justice and territorial democracy, starting with the transformation of social representations and self-representations of elderly people. This brings again to the fore the question of space—both in the form of public and third space—and the essential role of cities in offering opportunities for a high-level quality of life, especially to those less-advantaged citizens that cannot pay for it for themselves. A second principle of urban policy is strictly linked with the former. This concerns the right to live in a high-quality urban space, wherever one lives. The segmentation of the city into privileged and high-quality areas (generally the real and symbolic centers of cities) with a large endowment of territorial services, on the one hand, and deprived areas (generally the suburbs), on the other, is to be avoided. The principle of territorial democracy is the basis of a full implementation of the right to a high-quality life without having to move one’s place of residence.

^2^The main trends associated with population ageing are an increase in the segment of “third age” tourists, the formation of a new segment of “senior tourism,” the creation of high-tech hotel and resort complexes with robust geriatric care to promote active ageing, and, of course, the widespread adoption of the technology, Telehealth and Telecare, to support the aspirations of older people to travel and continue their active lifestyles (Nikitina and Vorontsova, 2015).

^3^The imaginary of places, either collective or individual, is affected by literary narrations, those deriving from journalism and travel magazines, films, and, more and more, from the stories and photos posted online by those who visit those places and who talk about them on their social networks.

^4^For this purpose, there has been a growing spread of “roots tourism,” increasingly supported by the numerous associations dedicated to this end and also by institutional initiatives (Carrera, 2021).

^5^“The older and their desire for experience. One of the new challenges of tourism,” International Conference Unicartourism2, REALD University, Vlorë (Albania) (22–23 April 2023) (Carrera, 2022).

^6^For example, the Hilton London Metropole Hotel is interested in such developments and plans to create special conditions for the recreation of older people with both the Telehealth and the Telecare services. Senior tourists are now more technologically savvy than they used to be two decades ago. In addition, their use of ICT reflects their confidence and independence in traveling. Nevertheless, since senior tourism is a new developing branch of tourism, much more research has to be done, including research into the use of ICT by these older travelers.

^7^Moscardo observes that, as noted by Fleischer and Pizam (2002), few reviews distinguish between studies that have profiled seniors only and those where seniors have been compared to other groups. This review suggests that senior travelers stay longer at their destination, spend more time planning, and visit friends and relatives more often. In addition, there is some evidence that seniors are attracted to certain types of travel. Pearce and Lee (2005), for example, describes the phenomenon of long-distance driving often associated with lengthy stays in warmer locations during winter. Fleischer and Pizam (2002) report strong associations between senior travelers and package tours, especially motorcoach and cruise ship options (Moscardo, 2006, p. 32). While it is quite common for authors to claim that present and future senior travelers will be wealthier, healthier, more active, and have more time than their predecessors (Fleischer and Pizam, 2002; Kim et al., 2003), very little empirical evidence is available on the characteristics that distinguish senior travelers from other age groups.

^8^The interviews were mostly conducted in person by the author of the article, with only a few conducted over the phone (19), due to the unavailability of the interviewees to be heard in person. It is worth noting that these subjects are mainly attributable to the types of Standby and Motionless.

^9^The territorial scope of reference was that of the Puglia region in southern Italy, and those indicated are the six provinces.

^10^In a semi-directed interview the interventions do not have a predefined order, and so sensitivity and flexibility are needed to adapt the use of a trace to the meaning frameworks of the respondents.
